# Glucoraphanin Triggers Rapid Antidepressant Responses in a Rat Model of Beta Amyloid-Induced Depressive-like Behaviour

**DOI:** 10.3390/ph15091054

**Published:** 2022-08-26

**Authors:** Paolo Tucci, Maria Bove, Vladyslav Sikora, Stefania Dimonte, Maria Grazia Morgese, Stefania Schiavone, Lorenzo Di Cesare Mannelli, Carla Ghelardini, Luigia Trabace

**Affiliations:** 1 Department of Clinical and Experimental Medicine, University of Foggia, 71122 Foggia, Italy; 2 Department of Pathology, Sumy State University, 40007 Sumy, Ukraine; 3 Pharmacology and Toxicology Section, Department of Neuroscience, Psychology, Drug Research and Child Health (NEUROFARBA), University of Firenze, 50139 Firenze, Italy

**Keywords:** glucoraphanin, antidepressant, amyloid beta

## Abstract

Glucoraphanin (GRA) is a natural compound that has shown beneficial effects in chronic diseases and in central nervous system disorders. Moreover, GRA displayed antidepressant activity in preclinical models. We have previously demonstrated that a single intracerebroventricular administration of soluble amyloid-beta 1-42 (sAβ 1-42) in rat evokes a depressive-like phenotype by increasing immobility frequency in the forced swimming test (FST). The aim of this work was to investigate the effect of GRA in naïve and in sAβ-1-42-treated rats by using the FST. Behavioural analyses were accompanied by neurochemical and biochemical measurements in the prefrontal cortex (PFC), such as serotonin (5-HT), noradrenaline (NA), kynurenine (KYN), tryptophan (TRP), reactive oxygen species (ROS) and the transcription nuclear factor kappa B (NF-kB) levels. We reported that GRA administration in naïve rats at the dose of 50 mg/kg reduced the immobility frequency in the FST and increased 5-HT and NA levels in the PFC compared to controls. At the same dose, GRA reverted depressive-like effects of sAβ 1-42 administration, restored the 5-HT levels and reduced NF-kB, KYN and ROS levels in PFC. In conclusion, GRA rapidly reverting depressive-like behaviour, together with biochemical and neurochemical alterations, might represent a safe and natural candidate for the treatment of depression.

## 1. Introduction

The cruciferous plants (broccoli, cauliflower, cabbage, etc.) are highly popular foods because of their health effects, as evidenced in several studies. These effects are ascribed to the high content of glucosinolates that are converted, by both the plant enzyme myrosinase and by the microflora of the gastrointestinal tract, to isothiocyanates (active compounds) [1].

A large amount of glucoraphanin (GRA) (the glucosinolate of sulforaphane) has been found in *Brassica oleracea*, with 10–100 times higher levels in 3-day-old sprouts [2]. After ingestion, the GRA is converted to the isothiocyanate sulforaphane (SFN) by the myrosinase enzyme activity of enteric microflora [1]. Together with its anticancer effects in animal and cellular models, this compound has also shown beneficial effects in other chronic diseases and in central nervous system (CNS) disorders. In a placebo-controlled, double-blind, randomized clinical trial involving young individuals (age 13–27 years) with autism spectrum disorder, SFN reverted different behavioural dysfunctions improving social interaction, abnormal behaviour and verbal communication [3]. Moreover, a recent study reported the beneficial effects of SFN on autism spectrum disorder biochemical alterations, such as redox dysregulation, heat shock response and immune dysfunctions in peripheral blood mononuclear cells [4]. In addition, SFN showed an improvement of cognitive functions in a small cohort of patients with schizophrenia [5].

Although available clinical trials are limited, there is a large number of preclinical studies. It has been reported that the SFN has protective effects in different animal models of Parkinson’s [6], Huntington’s [7] and Alzheimer’s diseases [8]. In particular, the administration of SFN in a mouse model of AD induced by amyloid beta ameliorated cognitive functions in the Y-maze and in the passive avoidance behavioural tests.

Several lines of evidence highlighted that there is a high incidence of depression among patients with Alzheimer’s disease (AD) before symptoms occur. Moreover, some forms of depression seem to be linked to the increase in amyloid beta (Aβ) levels, without plaque deposition, independently of the underlying AD brain pathology [9]. In this context, we have previously demonstrated that a single intracerebroventricular (i.c.v.) administration of soluble amyloid-beta 1-42 (sAβ 1-42) in rat evokes a depressive-like phenotype with depressive-like behaviour in the forced swimming test (FST), reduction in serotonin (5-HT) levels in the prefrontal cortex (PFC) [10,11] and a decrease in plasmatic corticosterone concentrations [12]. Moreover, the antidepressant fluoxetine was able to decrease plasmatic levels of sAβ 1-42 [13]. Interestingly, GRA and SFN were reported to have antidepressant effects in a mice model of depression. Indeed, repeated SFN administration significantly decreased the immobility time in the FST and tail suspension test (TST) [14].

The same results were retrieved in mice exposed to another behavioural paradigm known to induce depressive-like outcomes, such as the chronic mild stress paradigm [14]. Moreover, pretreatment of mice with SFN and dietary intake of food containing 0.1% GRA during juvenile and adolescent stages prevented the depressive-like phenotype induced after repeated social defeat stress [15].

Although the etiology of the depressive symptomatology is not fully understood, dysregulation of the central serotonergic and noradrenergic systems may be primarily involved [16]. Moreover, a number of studies associated depressive state and inflammation, involving the kynurenine (KYN) metabolism pathway [17]. Indeed, the KYN pathway is one of the two metabolic pathways of tryptophan (TRP), together with the 5-HT pathway [18]. Recently, it has been reported that amyloid beta upregulated the KYN pathway in neurons and that indoleamine 2,3 dioxygenase (IDO) inhibitors exhibit neuroprotective effects in animal models of AD [19,20,21]. The IDO inhibitors ameliorated behaviours associated with increased anxiety in the elevated plus maze and depressive-like behaviours in the TST [22].

Hence, the aim of this work was to investigate the effect of GRA in naïve and in sAβ 1-42-treated rats by using the FST, a widely used test to assess the capacity of antidepressant agents to switch passive behaviours in active forms of coping [23]. We evaluated in the prefrontal cortex (PFC), the effects of GRA treatment on serotonergic and noradrenergic neurotransmissions, as well as the possible involvement of KYN and TRP alterations.

Considering that sAβ 1-42 administration activates the inflammatory pathway and induces dysfunctions of antioxidant pathways [24,25,26], we also evaluated the reactive oxygen species (ROS) levels and the transcription nuclear factor kappa B (NF-kB) that serves as a pivotal mediator of inflammatory responses. The treatment and doses of GRA were chosen according to [14,27].

## 2. Results

### 2.1. Effects of GRA on Depressive-like Behaviour in the FST

**Figure 1:**
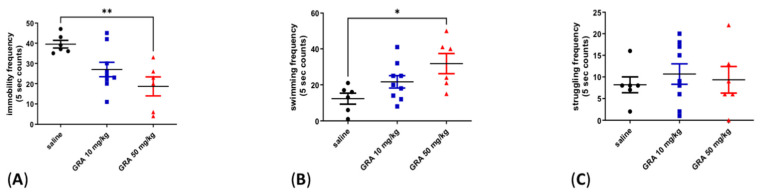
Effect of GRA administration in the FST in naïve rats. Effect of GRA administration on immobility (**A**), swimming (**B**) and struggling (**C**) frequencies in the FST in rats treated (i.p.) with saline (n = 6, black dots), GRA 10 mg/kg (n = 9, blue dots) and GRA 50 mg/kg (n = 6, red dots). * *p* < 0.05, ** *p* < 0.01 GRA 50 mg/kg vs. saline. One-way ANOVA followed by Tukey’s post hoc test.

GRA treatment at a dose of 50 mg/kg i.p. was able to reduce the immobility frequency compared to vehicle, whereas GRA treatment at a dose of 10 mg/kg i.p. did not affect immobility (Figure 1A, one-way ANOVA F = 7.108 followed by Tukey’s post hoc test, *p* < 0.01, GRA 50 mg/kg vs. saline). Consequently, GRA 50 mg/kg i.p., but not 10 mg/kg i.p., increased swimming frequency (Figure 1B, one-way ANOVA F = 0.4939 followed by Tukey’s post hoc test, *p* < 0.05 GRA 50 mg/kg vs. saline). The two GRA doses did not affect the struggling frequency (Figure 1C, one-way ANOVA F = 0.2637 followed by Tukey’s post hoc test, n.s.).

### 2.2. Effects of GRA on Cortical 5-HT and Noradrenaline (NA) Levels

**Figure 2:**
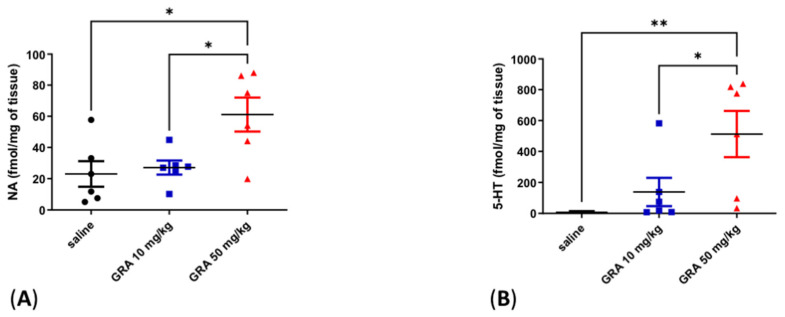
Effect of GRA administration on cortical monoamine quantifications in naïve rats. Effect of GRA administration on cortical NA (**A**) and 5-HT (**B**) (n = 6 per group) in rats treated (i.p.) with saline (black dots), GRA 10 mg/kg (blue dots) and GRA 50 mg/kg (red dots). * *p* < 0.05, ** *p* < 0.01 GRA 50 mg/Kg vs. saline. * *p* < 0.05, GRA 50 mg/kg vs. GRA 10 mg/kg. One-way ANOVA followed by Tukey’s post hoc test.

Cortical 5-HT and NA levels were significantly increased in animals treated with GRA 50 mg/kg compared to GRA 10 mg/kg and control rats (Figure 2A, one-way ANOVA F = 6.369 followed by Tukey’s multiple comparison test, *p* < 0.05 GRA 50 mg/kg vs. saline, *p* < 0.05 GRA 50 mg/kg vs. GRA 10 mg/kg; Figure 2B one-way ANOVA F = 6.618 followed by Tukey’s multiple comparison test, *p* < 0.01 GRA 50 mg/kg vs. saline, *p* < 0.05 GRA 50 mg/kg vs. GRA 10 mg/kg).

### 2.3. Effects of GRA on sAβ 1-42-Induced Depressive-like Behaviour in the FST

**Figure 3:**
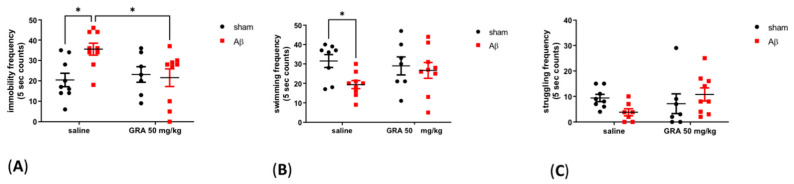
Effect of GRA administration in the FST in sAβ-1-42-treated rats. Effect of GRA (50 mg/kg) administration on immobility (**A**), swimming (**B**) and struggling (**C**) frequencies (saline sham n = 9, saline sAβ 1-42 n = 9, GRA sham n = 7 and GRA sAβ 1-42 n = 9) in the FST in rats 7 days after i.c.v. injection of vehicle (sham, 5 µL, black dots) or Aβ (Aβ, 4 µM, red dots). * *p* < 0.05 saline sAβ 1-42 vs. saline sham; * *p* < 0.05 GRA sAβ 1-42 vs. saline sAβ 1-42. Two-way ANOVA followed by Bonferroni’s post hoc test.

From a behavioural point of view, as showed in Figure 3A, results reported an increase in the immobility frequency in sAβ 1-42-injected animals compared to sham-operated rats (Two-way ANOVA F = 3.43 followed by Bonferroni’s post-hoc test, *p* < 0.05 saline sAβ 1-42 vs. saline sham). Moreover, in sAβ 1-42-treated rats, GRA 50 mg/kg administration induced a significant immobility decrease in the FST compared to animals that received only sAβ 1-42 (Figure 3A, Two-way ANOVA F = 5.229 followed by Bonferroni’s post-hoc test, *p* < 0.05 GRA sAβ 1-42 vs. saline sAβ 1-42 saline). As regarding swimming frequency, we found a significant decrease in sAβ 1-42-treated group compared to sham control (saline) rats (Figure 3B, Two-way ANOVA F = 4.112 followed by Bonferroni’s post-hoc test, *p* < 0.05 saline sAβ 1-42 vs. saline sham). The treatment did not affect the struggling frequency (Figure 3C, Two-way ANOVA F = 3.378 followed by Bonferroni’s post-hoc test, n.s.). Since FST might be influenced by motor strength or endurance impairments, during the habituation period we checked motor functions and we did not noticed any apparent motor deficits (data not shown). Treatments did not affect the locomotor behaviour of animals in the open field test (data not shown). 

### 2.4. Effects of GRA on Cortical 5-HT and NA Levels in sAβ-1-42-Treated Animals

**Figure 4:**
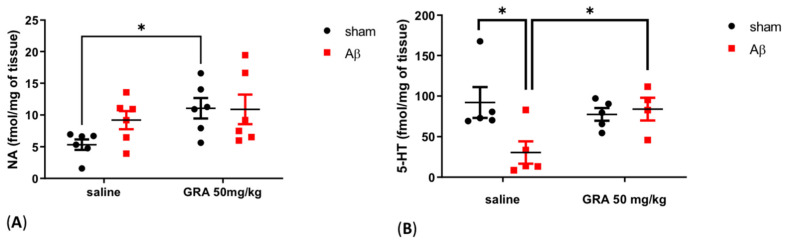
Effect of GRA administration on cortical monoamine quantifications in sAβ-1-42-treated rats. Effect of GRA (50 mg/kg) administration on cortical NA ((**A**), n = 6 per group) and 5-HT ((**B**), saline sham n = 5, saline sAβ 1-42 n = 5, GRA sham n = 5 and GRA sAβ 1-42 n = 4) in rats 7 days after i.c.v. injection of vehicle (sham, 5 µL, black dots) or Aβ (Aβ, 4 µM, red dots). * *p* < 0.05 GRA sham vs. saline sham; * *p* < 0.05 saline sAβ 1-42 vs. saline sham; * *p* < 0.05 GRA sAβ 1-42 vs. saline sAβ 1-42. Two-way ANOVA followed by Bonferroni’s post hoc test.

In sham rats receiving GRA 50 mg/kg injection, cortical NA content was increased compared to sham control animals (Figure 4A, two-way ANOVA F = 5.117 followed by Bonferroni’s multiple comparison test, *p* < 0.05 GRA sham vs. saline sham-operated rats), while no differences were found in sAβ-1-42-treated groups (Figure 4A, two-way ANOVA F = 1.507 followed by Bonferroni’s multiple comparison test, n.s.).

In Figure 4B, sAβ-1-42-treated animals showed a decrease in cortical 5-HT levels compared to sham-operated rats (two-way ANOVA F = 3.688 followed by Bonferroni’s multiple comparison test, *p* < 0.05 saline sAβ 1-42 vs. saline sham-operated rats). Moreover, in sAβ 1-42 animals, GRA 50 mg/kg administration was able to increase cortical 5-HT compared to controls (Figure 4B, two-way ANOVA F = 5.608 followed by Bonferroni’s multiple comparison test, *p* < 0.05 GRA sAβ 1-42 vs. saline sAβ 1-42).

### 2.5. Effects of GRA on TRP and KYN Levels in sAβ-1-42-Treated Animals

**Figure 5:**
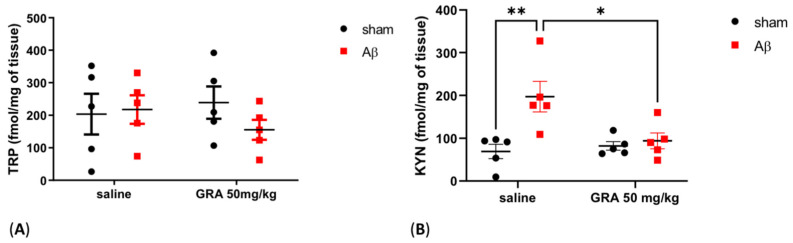
Effect of GRA administration on cortical TRP and KYN quantifications in sAβ-1-42-treated rats. Effect of GRA (50 mg/kg) administration on cortical TRP ((**A**), n = 5 per group) and KYN ((**B**), n = 5 per group) in rats 7 days after i.c.v. injection of vehicle (sham, 5 µL, black dots) or Aβ (Aβ, 4 µM, red dots). ** *p* < 0.01 saline sAβ 1-42 vs. saline sham; * *p* < 0.05 GRA sAβ 1-42 vs. saline sAβ 1-42. Two-way ANOVA followed by Bonferroni’s post hoc test.

In order to evaluate whether 5-HT alterations were linked to TRP metabolism alteration, we measured cortical KYN and TRP levels. TRP concentrations did not show any difference among experimental groups (Figure 5A, Two-way ANOVA F = 1.027 followed by Bonferroni’s multiple comparisons test, n.s.), while we found that KYN levels were significantly increased in rats treated with sAβ 1-42 compared to sham-operated animals (Figure 5B, Two-way ANOVA F = 9.902 followed by Bonferroni’s multiple comparisons test, *p* < 0.01 saline sAβ 1-42 vs. saline sham). Finally, the KYN levels were restored at the levels of control groups in sAβ 1-42-injected rats treated with GRA 50 mg/kg (Figure 5B, Two-way ANOVA F = 4.131 followed by Bonferroni’s multiple comparisons test; *p* < 0.05 GRA sAβ 1-42 vs. saline sAβ 1-42).

### 2.6. Effects of GRA on Cortical ROS Content and NF-kB Levels in sAβ-1-42-Treated Animals

**Figure 6:**
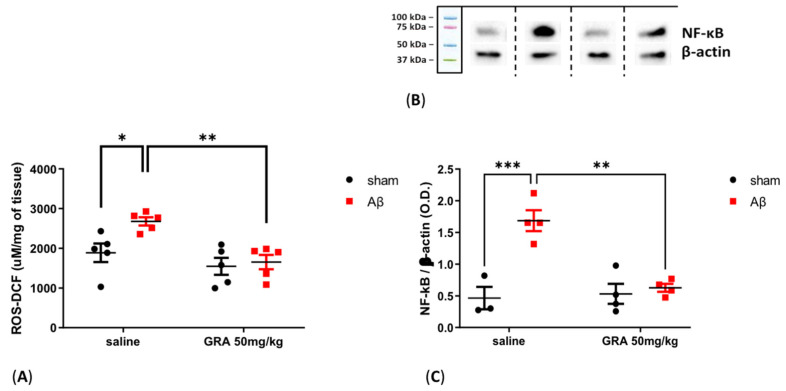
Effect of GRA administration on cortical ROS measurement and NF-kB protein expression levels in sAβ-1-42-treated rats. Effect of GRA (50 mg/kg) administration on cortical ROS content ((**A**), n = 5 per group), NF-kB ((**C**), saline sham n = 3, saline sAβ 1-42 n = 4, GRA sham n = 4 and GRA sAβ 1-42 n = 4) in rats 7 days after i.c.v. injection of vehicle (sham, 5 µL, black dots) or Aβ (Aβ, 4 µM, red dots). (**B**). Representative images of Western blotting bands of NF-kB and β-actin housekeeping gene in the prefrontal cortex of saline sham, saline sAβ 1-42, GRA sham and GRA sAβ 1-42 -treated animals. *** *p* < 0.001, * *p* < 0.05 saline sAβ 1-42 vs. saline sham; ** *p* < 0.01 GRA sAβ 1-42 vs. saline sAβ 1-42. Two-way ANOVA followed by Bonferroni’s post hoc test.

As reported in Figure 6, ROS content was increased in the PFC of sAβ-1-42-injected animals compared to sham-operated rats (two-way ANOVA F = 5.612 followed by Bonferroni’s post hoc test, *p* < 0.05 saline sAβ 1-42 vs. saline sham). Moreover, in sAβ-1-42-treated rats, GRA 50 mg/kg administration induced a significant reduction in cortical ROS levels compared to rats receiving only sAβ 1-42 (Figure 6A, two-way ANOVA F = 13.02 followed by Bonferroni’s post hoc test, *p* < 0.01 GRA sAβ 1-42 vs. saline sAβ 1-42). Concerning cortical NF-kB levels, there was an increase in sAβ-1-42-treated animals compared to control rats (two-way ANOVA F = 20.45 followed by Bonferroni’s post hoc test, *p* < 0.001 saline sAβ 1-42 vs. saline sham), while, in sAβ-1-42-injected animals, GRA 50 mg/kg administration induced a significant reduction in cortical NF-kB levels compared to control (Figure 6B, 6C, two-way ANOVA F = 11.59 followed by Bonferroni’s post hoc test, *p* < 0.01 GRA sAβ 1-42 vs. saline sAβ 1-42).

## 3. Discussion

In this study, we showed that GRA administration at the dose of 50 mg/kg reduced the immobility behaviour in the FST in naïve rats and reverted depressant effects of central sAβ 1-42 administration, mimicking the effects of antidepressants [13]. Over recent decades, the pharmacotherapy for major depressive disorders has been focused on the modulation of monoaminergic neurotransmissions and the use of selective serotonin reuptake inhibitors (SSRIs) has widely increased. Interestingly, we found that the GRA at the dose of 50 mg/kg increased 5-HT levels in the PFC of naïve rats and restored the cortical 5-HT levels to the control levels in sAβ-1-42-treated rats. GRA was also able to inhibit cortical increase in KYN levels, suggesting a TRP switch toward the 5-HT pathway that counteracts depressive behaviour [28,29]. The TRP concentrations were not different among experimental groups, confirming the shift from KYN to 5-HT synthesis.

The KYN is produced by TRP, instead of 5-HT, when proinflammatory agents stimulate IDO enzymes located in the brain [18]. In our study, the sAβ 1-42 administration could induce IDO directly [30] and indirectly, increasing NF-kB levels as well as pro-oxidant and inflammatory agents [31]. GRA and SFN inhibit IDO through KeaP1/NrF2 pathway activation [32], but the involvement of this pathway seems unlikely considering the quick (30 min after a single administration) induction of effect on rat behaviour and 5-HT and KYN levels [33]; this pathway could play a critical role in nourishing this effect over time as reported in mice that received a sub-chronic treatment with SFN [14]. However, we performed only one injection of GRA, 30 min before the FST trial, in order to evaluate the acute effect, without testing whether the effect lasts after 24 h. Thus, future work will study the duration of this acute treatment.

The acute and rapid effects of GRA and SFN have been matched with hydrogen sulfide (H_2_S) release [34]. Previous findings demonstrated that the isothiocyanates act as H_2_S-releasing compounds, and SFN and GRA showed an equivalent behaviour [35,36]. 

Endogenous H_2_S is synthesized and released immediately, and its role as the novel gaseous mediator has been recognized by a number of studies [37]. Its presence in major areas of human and rat brains has been demonstrated as well as the role in the pathogenesis of some neurodegenerative diseases [38]. Moreover, an antidepressant effect of H_2_S in rats [39] and an upregulation of 5-HT in the striatal area and PFC [40] induced by H_2_S have been reported.

The 5-HT increase observed in our experimental condition might follow the H_2_S release, and this requires an amount of TRP to subtract from the KYN synthesis pathway. We reported, as a consequence, a reduction in KYN levels.

Moreover, the NA increase levels in PFC of intact and sham-operated animals could be induced by a well-known pre-synaptic action of H_2_S [41].

The NA increase has been reported in sAβ-1-42-treated rats, reflecting a neuroprotective phenomenon and a compensatory mechanism [42,43]. Moreover, NA has a role as a redox cycling modulator and mild antioxidant [44], and this feature could explain the reduction in ROS levels that impact NF-kB protein levels [45,46]. Indeed, the NF-kB pathway is fine-tuned by the redox environment and responds on different timescales [47,48]. The adrenergic activation eventually could stimulate the activity of many ubiquitin ligases [49,50]. The reduction in NF-kB protein levels reported here might be caused by an accelerated clearance through ubiquitin–proteasome system (that can be stimulated by SFN in brain and peripheral tissues [51,52]) as the first step in stopping the inflammatory activity. Our results have shown a different activity of GRA in comparison to SFN that inhibited the NF-kB DNA binding [46].

## 4. Materials and Methods

### 4.1. Animals

The present experimental study was conducted on a male Wistar rats model (250–275 g, 8-weeks-old, Harlan, S. Pietro al Natisone, Udine, Italy). Rodents were kept under group housing conditions (four per cage), controlled 12h light/dark cycle (lights on from 7:00 AM to 7:00 PM), acceptable room temperature (22 ± 1°C) and humidity (55 ± 5%) levels. Rats had *ad libitum* access to standard pellets and tap water. Monitoring of animals’ welfare was performed daily throughout the entire experimental period. All efforts were made to minimize the pain, suffering and distress experienced by the animal, and reduce their number.

### 4.2. Surgery and sAβ 1-42 Infusion

The sAβ 1-42 peptide was purchased from Tocris (Bristol, UK). The stock solution was freshly prepared by using sterile double distilled water (vehicle) at a concentration of 4 μM.

The rats were anesthetized, placed in a stereotaxic frame, and immobilized by blunt ear bars (David Kopf Instruments, Tujunga, CA, USA). Immediately after the scalp was shaved from the wool and disinfected. Using a sterile scalpel, the operator cut the skin over the midline of the rat head to expose the skull. Herein, a smaller-diameter hole was drilled to insert the very thin injection needle (30-gauge stainless steel tubing; Cooper’s Needles, Birmingham, UK).

Coordinates for i.c.v. infusions were based on [53]: AP = −0.5, ML = +1.2 and DV = −3.2 from bregma, with the incisor bar set at -3.3 mm. Animals received sAβ 1-42 (5 μL) through a 25 μL Hamilton microsyringe. The infusion rate of 2 μL/min over a period of 2.5 min was controlled by a microsyringe pump. The injection needle was left in place for 5 min prior to removal. The skin was closed using an absorbable Vicryl suture.

Control rats were infused with vehicle only, because reverse soluble sAβ 42-1, used in preliminary experiments, had no effect on the measured parameters and was indistinguishable from vehicle alone (unpublished observations). The post injection placement of the needle track was confirmed at the time of dissection. Next experimental procedures were performed 7 days after i.c.v. administration (sham-operated or soluble sAβ 1-42 -treated groups).

### 4.3. Pharmacological Treatments and Experimental Design

GRA was purified from Tuscan black kale seeds at the Bologna laboratory (CREA-AA; previously CRA-CIN) according to an established method [54], dissolved in saline (vehicle) and given intraperitoneally (i.p.) 30 min before the behavioural performance assessment in the FST (test phase).

### 4.4. Forced Swimming Test

The protocol has been based on procedures previously described by [55]. 

The procedure was carried out in two sessions: pre-test session (15 min) and, twenty-four hours later, the test session (5 min). During the pre-test session, rats were separated and individually placed in a clear Perspex cylinder (70 cm height × 20 cm diameter) containing tap water to a depth of 30 cm. The water temperature was maintained constant at 25 °C during the procedure. Then after, the operator removed the animals from the cylinder, towel-dried in a clean plexiglass cage and then returned them to the home cage. Between each trial, the water was changed and the cylinders were cleaned. The test phase was performed twenty-four hours later, where each rat was checked under an identical method for 5 min. To video record the test phase, a video camera was set up on a tripod at a 90° angle to the cylinder. The analysis of these recordings was performed by observer-blind to the experimental model, who scored the following behaviours: rat floating that make only the necessary movements to keep its nose above the water (immobility), rat movement of limbs around the cylinder (swimming) and rat attempting to escape from cylinder (struggling). Immobility, swimming and struggling frequency has been counted every 5 s during the 5 min test session.

### 4.5. Post Mortem Tissue Analyses

Thirty minutes after GRA treatment, another batch of rats was euthanized and brains were immediately removed, collecting them at the same time for all the ex vivo assays. The brains were placed dorsal side up in an ice chilled rat brain matrix (World Precision Instruments, Sarasota, FL, USA) with slits spaced at 1 mm using an ice-chilled razor blade. Thereafter, PFC was immediately removed, accurately weighed with digital scales, rapidly frozen in liquid nitrogen, collected and stored at −80 °C refrigerator until neurotransmitters quantification was carried out. At the time of analysis, PFC tissue samples were homogenized in 10 volumes (w/V) of 0.1 N perchloric acid. The homogenates were stored on ice for 30 min and then centrifuged at 10,000× *g* for 10 min at 4 °C. Finally, the supernatants were then filtered with a millipore filter membrane, diluted and analyzed using high-performance liquid chromatography analysis (HPLC).

### 4.6. Neurochemical Analyses

NA, 5-HT and KYN concentrations were determined in the PFC by HPLC coupled with an electrochemical detector (Ultimate 3000RS -ECD, Dionex, Thermo Fisher Scientific, Horsham, UK), as previously published [56]. The separation was performed by a LC18 compressed cartridge column using reversed-phase conditions (Kinetex, 15 cm × 4.6 mm × 5 µm; Phenomenex, Bologna, Italy). The detection was accomplished by a thin-layer amperometric cell (Dionex, ThermoScientifics, Milan, Italy) with a 5 mm diameter glassy carbon electrode at a working potential of 0.400 or 750 V (KYN) vs. Palladium. The mobile phase consisted of an aqueous buffer containing 75 mM NAH_2_PO_4_, 1.7 mM octane sulfonic acid, 0.3 mM EDTA, acetonitrile 10%, buffered at pH 3.00. The flow rate was maintained by an isocratic pump (Shimadzu LC-10 AD, Kyoto, Japan) at 0.7 mL/min. Data were acquired and integrated by using Chromeleon software (version 6.80, Dionex, Thermo Fisher Scientific, San Donato Milanese, Italy).

### 4.7. ROS Measurement

ROS content was measured in PFC as previously described (33917814). Briefly, the tissue was homogenized in PBS 1X (pH = 7.4), and the fluorogenic dye 2′-7′dichlorofluorescein diacetate (Sigma Aldrich, Milano, Italy) at a final concentration of 5 mM was added to homogenized samples. Then, samples were incubated at 37 °C for 15 min and centrifuged at 4 °C, 12,500 rpm, for 10 min. The pellet was resuspended in 5 mL of PBS 1X and put on ice for 10 min. After 1 h of incubation at 37 °C, samples were analyzed using a fluorometer (Filter Max F5, Multi-Mode Microplate Reader, excitation length 475 nm, emission length 535 nm), and results were expressed as µM DCF/mg of tissue.

### 4.8. Western Blotting

Proteins were quantified in cortical homogenates using the Pierce BCA Assay (Thermo Fisher Scientific, Cleveland, OH, USA). Total lysate protein (40 µg) was separated by 4–15% Mini-PROTEAN™ TGX Stain-Free™ Protein Gels, 15 wells (Bio-Rad Laboratories Inc, Segrate (MI), Italy) and transferred onto nitrocellulose membranes (Bio-Rad Laboratories Inc, Segrate (MI), Italy). Membranes were then put in a blocking buffer (SigmaAldrich, Milan, Italy) for 1 h at room temperature and incubated overnight at 4 °C with primary mouse monoclonal antibodies against β-actin 1:1000 dilution, ab8226, Abcam, Cambridge, UK) and rabbit polyclonal antibodies against NF-kB 1:1000 dilution, ab16502, Abcam, Cambridge, UK). Subsequently, membranes were incubated with specific secondary antibodies goat anti-mouse IgG H&L (HRP) (1:5000 dilution, ab205719, Abcam, Cambridge, UK) and goat anti-rabbit IgG H&L HRP-conjugated antibody (1:5000 dilution, ab6721, Abcam, Cambridge, UK). Protein bands were visualized using a chemiluminescent ECL reagent (Bio-Rad Laboratories Inc, Segrate (MI), Italy) and detected by the ChemiDoc MP system (Bio-Rad Laboratories Inc, Segrate (MI), Italy). Measurements of the bands’ optical densities normalized against β-actin bands were performed by using ImageJ software (version 1.52a (National Institutes of Health, Bethesda, MD, USA), https://imagej.nih.gov/ij/ (accessed on 7 September 2020)). 

### 4.9. Statistical Analyses

Graph Pad^®^ 6.0 (GraphPad Software, San Diego, CA) for Windows was used to perform statistical analyses. Behavioural and neurochemical data were tested for normality and then analyzed by using One-way ANOVA followed by Tukey’s multiple comparisons test or Two-way ANOVA followed by Bonferroni’s multiple comparisons test. Differences were considered statistically significant when *P*-value was less than 0.05. 

## 5. Conclusions

A number of studies with SFN collected pre-clinical evidence of the neuroprotective effect on AD pathophysiology [57]. Moreover, GRA reduced neuropathic pain in different animal models [36].

In this work, GRA improved behaviour in rats that was associated with depressive-like phenotypes. Considering the better chemical stability over the time in comparison to SFN [27], GRA could become a novel natural antidepressant compound.

## Data Availability

Data is contained within the article.
